# Exploiting the potential of the ubiquitin-proteasome system in overcoming tyrosine kinase inhibitor resistance in chronic myeloid leukemia

**DOI:** 10.1016/j.gendis.2023.101150

**Published:** 2023-10-26

**Authors:** Xudong Li, Wei Li, Yanli Zhang, Linping Xu, Yongping Song

**Affiliations:** a Department of Hematology, Affiliated Cancer Hospital of Zhengzhou University and Henan Cancer Hospital, Zhengzhou, Henan 450008, China; b Academy of Medical Sciences, Zhengzhou University, Zhengzhou, Henan 450052, China; c Department of Hematology, The First Affiliated Hospital of Zhengzhou University, Zhengzhou, Henan 450052, China

**Keywords:** Chronic myeloid leukemia, Deubiquitinases, E3 ligase, PROTAC, TKI resistance, Ubiquitin-proteasome system

## Abstract

The advent of tyrosine kinase inhibitors (TKI) targeting BCR-ABL has drastically changed the treatment approach of chronic myeloid leukemia (CML), greatly prolonged the life of CML patients, and improved their prognosis. However, TKI resistance is still a major problem with CML patients, reducing the efficacy of treatment and their quality of life. TKI resistance is mainly divided into BCR-ABL-dependent and BCR-ABL-independent resistance. Now, the main clinical strategy addressing TKI resistance is to switch to newly developed TKIs. However, data have shown that these new drugs may cause serious adverse reactions and intolerance and cannot address all resistance mutations. Therefore, finding new therapeutic targets to overcome TKI resistance is crucial and the ubiquitin-proteasome system (UPS) has emerged as a focus. The UPS mediates the degradation of most proteins in organisms and controls a wide range of physiological processes. In recent years, the study of UPS in hematological malignant tumors has resulted in effective treatments, such as bortezomib in the treatment of multiple myeloma and mantle cell lymphoma. In CML, the components of UPS cooperate or antagonize the efficacy of TKI by directly or indirectly affecting the ubiquitination of BCR-ABL, interfering with CML-related signaling pathways, and negatively or positively affecting leukemia stem cells. Some of these molecules may help overcome TKI resistance and treat CML. In this review, the mechanism of TKI resistance is briefly described, the components of UPS are introduced, existing studies on UPS participating in TKI resistance are listed, and UPS as the therapeutic target and strategies are discussed.

## Background

Chronic myeloid leukemia (CML) is a unique type of leukemia first discovered in the 1840s, accounts for about 15% of adult leukemia cases, and is characterized by uncontrolled growth of myeloid cells.^1^, ^2^, ^3^ According to a systematic analysis from 1990 to 2017, 34,179 CML cases were reported globally in 2017 with 24,054 deaths.^4^ At present, sufficient studies have confirmed that the Philadelphia (Ph) chromosome, a short chromosome produced by the reciprocal translocation between the breakpoint cluster region (BCR) gene and the ABL1 kinase Abelson (ABL) gene located between chromosomes 9 and 22, is the only driving factor for CML.^5^ The fusion gene can encode and activate chimeric BCR-ABL protein, and then exert its tyrosine kinase function which affects the downstream signaling pathways, leading to leukemia.^6^

Tyrosine kinase inhibitors (TKIs) are a current treatment agent that has shown outstanding anti-CML effects. From the first generation TKI imatinib to the first novel TKI targeting ABL myristoyl pocket (STAMP inhibitor) asciminib, the treatments have reduced the burden of disease more effectively and faster than other treatments. TKI agents also provide a large number of choices for patients and greatly reduce the number of patients who develop accelerated or blast phase disease.^7^^,^^8^ Although a number of TKIs have emerged, many patients still fail to respond to treatment because of TKI resistance, mainly driven by BCR-ABL-dependent and independent resistance.^9^ The presence of drug resistance and treatment failure warrant the search for more targets in CML therapy.

Ubiquitin proteasome system (UPS) is the main regulatory factor targeting protein degradation in eukaryotic cells. It plays an important role in maintaining the balance of protein turnover *in vivo* and affects a wide range of biological processes.^10^ In the field of hematological oncology, UPS has shown its therapeutic potential. Bortezomib, a selective inhibitor of the 26S proteasome derived from the UPS, has become the first-line combination therapy for multiple myeloma and mantle cell lymphoma.^11^ Moreover, a few components of UPS, such as E1/E2/E3 ligase, deubiquitinases (DUBs), proteasome inhibitors, and ubiquitin-like proteins, play an important role in the occurrence and development of many other hematological tumors. As a protein degrader, UPS may be a good potential candidate in the treatment of CML and is expected to effectively overcome TKI resistance to improve the prognosis of CML patients.

In this review, the types and mechanisms of TKI resistance in CML treatment are systematically described, and the composition of UPS and the process of protein degradation are introduced. Based on the literature, a classified introduction of the various components of UPS that participate in the occurrence and development of CML and TKI resistance is provided. Additionally, potential targets to overcome TKI resistance and treat CML are summarized. As the most cutting-edge technology related to UPS, the prospect of PROteolysis TArgeting Chimeras (PROTAC) technology is also reviewed.

### A brief introduction to TKI resistance

Since the 1990s, the successful development of TKI targeting BCR-ABL has ushered in the era of small molecule targeted drugs.^12^ Up to 2023, the U.S. Food and Drug Administration has approved five types of TKIs for CML, including the first-generation imatinib, the second-generation dasatinib, nilotinib, and bosutinib, and the third-generation ponatinib. In 2019, China approved the second-generation TKI, flumatinib for the treatment of CML.^13^ Although the use of TKIs has greatly benefited patients, 20% of patients fail to achieve the desired effect and develop TKI resistance immediately after the initial beneficial effect is seen. Most of these patients also have a high probability of entering the acute phase or blast phase, resulting in treatment failure.^7^^,^^14^

**Figure 1:**
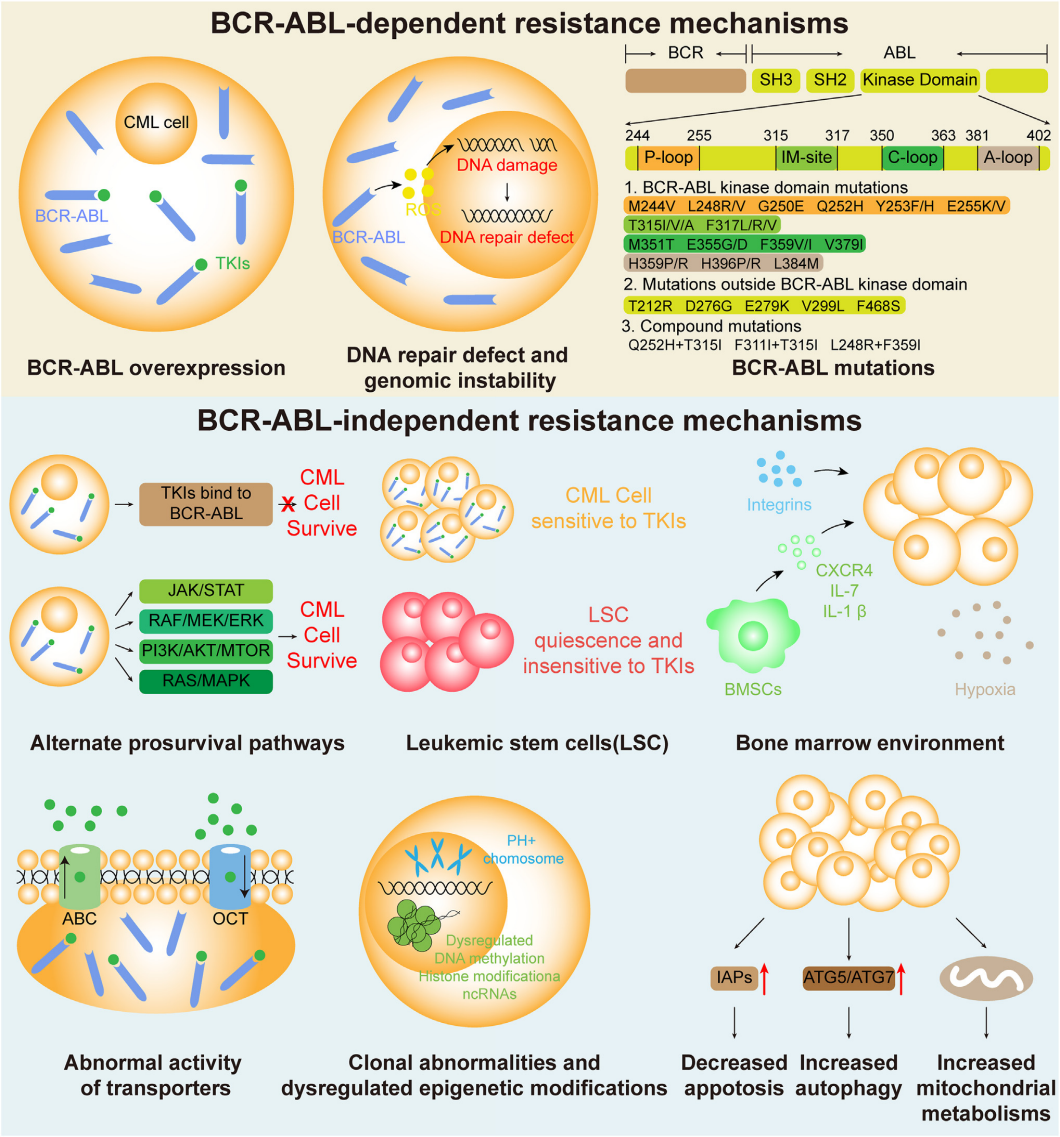
The mechanisms of TKI resistance. The mechanisms of TKI resistance are mainly divided into BCR-ABL-dependent and BCR-ABL-independent resistance. The BCR-ABL-dependent resistance includes BCR-ABL mutation (mutations of the BCR-ABL kinase domain, mutations outside the BCR-ABL kinase domains, and the compound mutations), BCR-ABL overexpression, DNA repair defect, and genomic instability. The BCR-ABL-independent resistance includes the activation of alternate prosurvival signaling pathways, leukemic stem cells (LSCs), bone marrow microenvironment, *etc*. TKI, tyrosine kinase inhibitor.

The mechanisms of TKI resistance are mainly divided into BCR-ABL-dependent resistance and BCR-ABL-independent resistance. The mechanisms of BCR-ABL-dependent resistance include BCR-ABL mutation, BCR-ABL overexpression, DNA repair defect, and genomic instability. The mechanisms of BCR-ABL-independent resistance include the activation of alternate prosurvival signaling pathways, leukemic stem cells (LSCs), and bone marrow microenvironment (Fig. 1).^9^^,^^15^

## BCR-ABL-dependent resistance

### BCR-ABL mutations

BCR-ABL mutations can be divided into mutations of the BCR-ABL kinase domain, mutations outside the BCR-ABL kinase domains, and compound mutations. Mutations of the BCR-ABL kinase domain are mutations in the ABL kinase domain or other kinase domains that change the conformation of BCR-ABL or completely hinder the binding of drugs to BCR-ABL, resulting in drug resistance. The mutation sites can be divided into four categories: (i) the ATP-binding P-loop, between amino acids (aa) 244 and 255; (ii) the imatinib mesylate direct binding site between aa 315 and 317; (iii) the C-loop (catalytic domain) between aa 350 and 363; and (iv) the A-loop (activation loop) between aa 381 and 402.^16^

The second and third-generation TKIs work by optimizing the affinity and binding sites of TKI to ABL kinase domains according to different mutation spectra. This mechanism is effective for most mutations except the T315I mutation, creating more drugs available for patients with TKI drug resistance. The T315I mutation, also known as the gatekeeper mutation, is the most common, occurring in 4% of imatinib mesylate-resistant CML patients, causing resistance to all second and third-generation TKIs.^9^ It has been reported that olverembatinib developed by Chinese pharmaceutical companies is effective for imatinib mesylate resistance. According to results of phase I and II clinical studies, olverembatinib monotherapy had good efficacy and tolerance in patients with CML-chronic phase and CML-acute phase patients, other TKI-tolerant CML-chronic phase and CML-acute phase patients as well as patients with T315I mutation.^17^, ^18^, ^19^

Mutations outside the BCR-ABL kinase domains and the compound mutations are uncommon. The former refers to the mutation of the SH2, SH3, and Cap domains which are involved in the automatic inhibition of ABL kinase. Compound mutations refer to two mutations within one BCR-ABL protein or two or more point mutations within several BCR-ABL proteins.^16^ It is reported that after ponatinib treatment, the secondary drug resistance mutations in CML patients with T315I mutations are defined as compound mutations based on T315I, including T315L, Q252H + T315I, and F311I + T315I, which challenges effective treatment.^20^

### BCR-ABL overexpression

CML genomic amplification or overexpression of BCR-ABL transcripts is often detected in patients with TKI resistance. A study demonstrated a relationship between BCR-ABL overexpression and BCR-ABL mutation, while in the absence of BCR-ABL amplification, mutations did not occur. This suggests that BCR-ABL overexpression may be an early manifestation of TKI resistance mutations, but more confirmatory evidence is needed.^21^

### DNA repair defect and genomic instability

Previous studies have shown that the repair of defective DNA and genomic instability caused by an imbalance of DNA damage response is related to the clonal evolution of CML. This evolution is an important promoting factor for the occurrence of TKI resistance and the malignant transformation of CML.^22^^,^^23^ In a retrospective study, 81 CML patients underwent targeted sequencing involving 386 genes. The results suggest that additional sex combs-like 1 mutation was the most common genetic damage in all stages of CML except ABL, followed by histone-lysine N-methyltransferase 2D and pyruvate carboxylase in CML-chronic phase, BCL6 corepressor like 1, and runt-related transcription factor 1 in CML-acute phase and runt-related transcription factor 1 and Wilms tumor 1 in CML-blastic crisis. These genetic damages are cumulative in the development of CML from chronic phase to blastic crisis, which may lead to poor prognosis.^24^

## BCR-ABL-independent resistance

### Activation of alternate prosurvival signaling pathways

Although TKIs inhibit the activity of BCR-ABL kinase, CML cells can activate alternative signaling pathways to maintain their survival. Alternative signaling pathways known to date are JAK (Janus kinase)/STAT (signal transducer and activator of transcription), RAF/MEK/extracellular signal-regulated kinase, PI3K (phosphoinositide 3-kinase)/AKT/mammalian target of rapamycin, RAS/mitogen-activated protein kinase, forkhead box O1, B cell leukemia/lymphoma 2, SRC, *etc*.^25^, ^26^, ^27^ From the treatment perspective, alternative signaling pathways are targeted to overcome drug resistance. A JAK inhibitor, ruxolitinib, when combined with TKI, was reported to reduce the phosphorylation of signal transducer and activator of transcription 3, promote CML cell death, and induced secondary remission in patients with TKI resistance.^28^

### Leukemic stem cells

LSCs are naturally insensitive to TKIs. TKI insensitivity is not only the root cause of TKI resistance in CML patients but also an important factor that challenges recovery and drug withdrawal.^29^ Although the survival of LSCs does not rely on the activities of BCR-ABL kinase, their survival is regulated by multiple signaling pathways (Wnt/β-catenin, Notch, transforming growth factor-beta), epigenetic regulatory protein (silent information regulator factor 2-related enzyme 1, protein arginine methyltransferase 5/7), transcriptional factors (p53, forkhead box M1, noncanonical nuclear factor-kappa B), cellular metabolic regulatory molecules (solute carrier 15 membrane proteins PEPT2) and other molecules.^30^, ^31^, ^32^, ^33^, ^34^, ^35^, ^36^, ^37^ The involvement of these molecules and pathways in LSCs' survival makes them potential targets for the eradication of LSCs. For example, venetoclax has demonstrated outstanding performance in the treatment of CML. Preclinical studies have shown that venetoclax combined with TKI effectively eradicated LSCs.^38^

### Bone marrow microenvironment

Abnormalities in the bone marrow microenvironment are closely related to the occurrence and development of leukemia. Cell–cell interaction mediated by various receptors in the bone marrow stroma, integrin participating in cell adhesion, hypoxic bone marrow environment, and cytokines, chemokines, and growth factors (C-X-C motif chemokine receptor 4, interleukin-17, interleukin-1β) secreted by stromal cells are involved in TKI resistance.^16^^,^^39^ Ravi Bhatia et al reported that changes in bone marrow stromal niches mediated by tumor necrosis factor alpha in CML enhanced the maintenance and growth of LSCs through C-X-C motif chemokine ligand 1–CXC chemokine receptor 2 signaling and inhibition of CXC chemokine receptor 2 can effectively eradicate CML LSCs and contribute to TKI resistance.^40^

### Other mechanisms

Except for activation of alternate prosurvival signaling pathways, LSCs, and bone marrow microenvironment, there are also other mechanisms of BCR-ABL-independent resistance such as the abnormal activity of transporters, clonal abnormalities, dysregulated epigenetic modifications, the up-regulation of inhibitors of apoptosis proteins (IAPs), and increased autophagy and mitochondrial metabolisms. Since the above mechanisms have been described in detail in some articles, we will not describe them too much here.^16^

## UPS

### A brief introduction to UPS

The UPS mediates the degradation of about 90% of proteins in organisms. Protein degradation is essential for regulating cell function and maintaining protein stability. The UPS consists of the ubiquitin (ub)-activating enzyme (E1), the ub-conjugating enzyme (E2), the ub-ligating enzyme (E3), ubiquitin, and the 26S proteasome.^41^^,^^42^

Ub is a highly conserved protein containing 76 amino acids with a molecular weight of 8.5 kDa. It contains seven lysine residues, namely K6, K11, K27, K29, K33, K48, and K63, which attach to substrate proteins in the form of single molecules or through specific heteropeptide bonds to form branched or forked polymer chains.^43^, ^44^, ^45^ The E1 enzyme is small in number and exists as two subtypes based on size, at 110 kDa and 117 kDa, which come from the same gene and mRNA.^46^ E2 on another hand is sized within 14–35 kDa and contains a conservative catalytic domain (ubiquitin-conjugating domain).^47^ There are about 40 E2s, which can be divided into four categories; class Ⅰ has only the ubiquitin-conjugating domain, while class Ⅱ and Ⅲ have N- or C-terminal domain respectively, and class IV contains both the N- and C-terminal domains, whereby the different domains affect the molecular weight and function of E2.^48^ Some E2s, such as ubiquitin-conjugating enzyme E2O and baculoviral IAP repeat containing 6, have both E2 and E3 activities. These E2s can directly bind to the substrate protein and mediate ubiquitin modification independent of other E3, called E2/E3 hybrid ubiquitin-protein ligase.^49^^,^^50^ The largest in number, there are over 600 E3 ubiquitin ligases that can specifically recognize and mediate the ubiquitination of substrate proteins. E3 ubiquitin ligases are divided into three categories: homologous with E6-associated protein C-terminus, really interesting new gene (RING), and ring-between-ring. E3s are the most important components in UPS, determining which proteins will be degraded.^51^, ^52^, ^53^ The 26S proteasome is a large (1500–2000 kDa) multi-subunit complex in the nucleus and cytoplasm of eukaryotic cells, including a catalytic core 20S proteasome and two regulatory subunits 19S proteasome. These proteasomes can recognize and degrade substrate proteins labeled by polyubiquitin chains.^10^

The process of ubiquitin can be reversed by specific deubiquitinases (DUBs), which balance the ubiquitination under the control of the E3 ubiquitin ligase and DUBs. There are more than 100 DUBs that are divided into seven categories: Ub C-terminal hydrolases (UCHs), ub-specific proteases (USPs), Machado-Josephin domain proteases, ovarian tumor proteases, motif interacting with Ub-containing novel DUB family, zinc-finger-containing ub peptidase, and Jab1/Mov34/MPN^+^ protease. The first six categories are cysteine-based DUBs and the last one belongs to zinc-binding metalloproteases.^54^

Ubiquitin-like proteins have been a hot topic in recent years. It is a group of proteins that are related to ub sequences and have a similar three-dimensional structure.^55^ They are mainly divided into two types: ubiquitin-like proteins (type I) and ubiquitin-like domain proteins (type II). The C-terminal of ubiquitin-like protein has one or two glycine residues and this is a characteristic sequence motif that is responsible for covalently linking to the substrates. Ubiquitin-like domain proteins lack C-terminal diglycine motifs and cannot bind to the target, but they can play a role in protein–protein interaction. At present, the ubiquitin-like proteins that can function as protein modifiers are autophagy-related 8/12, F locus adjacent transcript 10, fau and its ubiquitin-like domain, histone monoubiquitination 1, interferon-stimulated gene 15, neural precursor cell expressed developmentally down-regulated protein 8 (NEDD8), small ubiquitin-related modifier, ubiquitin-fold modifier 1, and ubiquitin related modifier 1. They are involved in important biological activities such as nuclear transport, proteolysis, translation, autophagy, and antiviral pathways.^56^^,^^57^

The types of ubiquitin modification include mono-ubiquitin, multi-site mono-ubiquitin, and polyubiquitin modifications. Polyubiquitin modification of K48 and K63 is the most studied. The former mainly mediates the degradation of substrate protein, while the latter mainly affects the localization and function of the substrate protein.^58^

**Figure 2:**
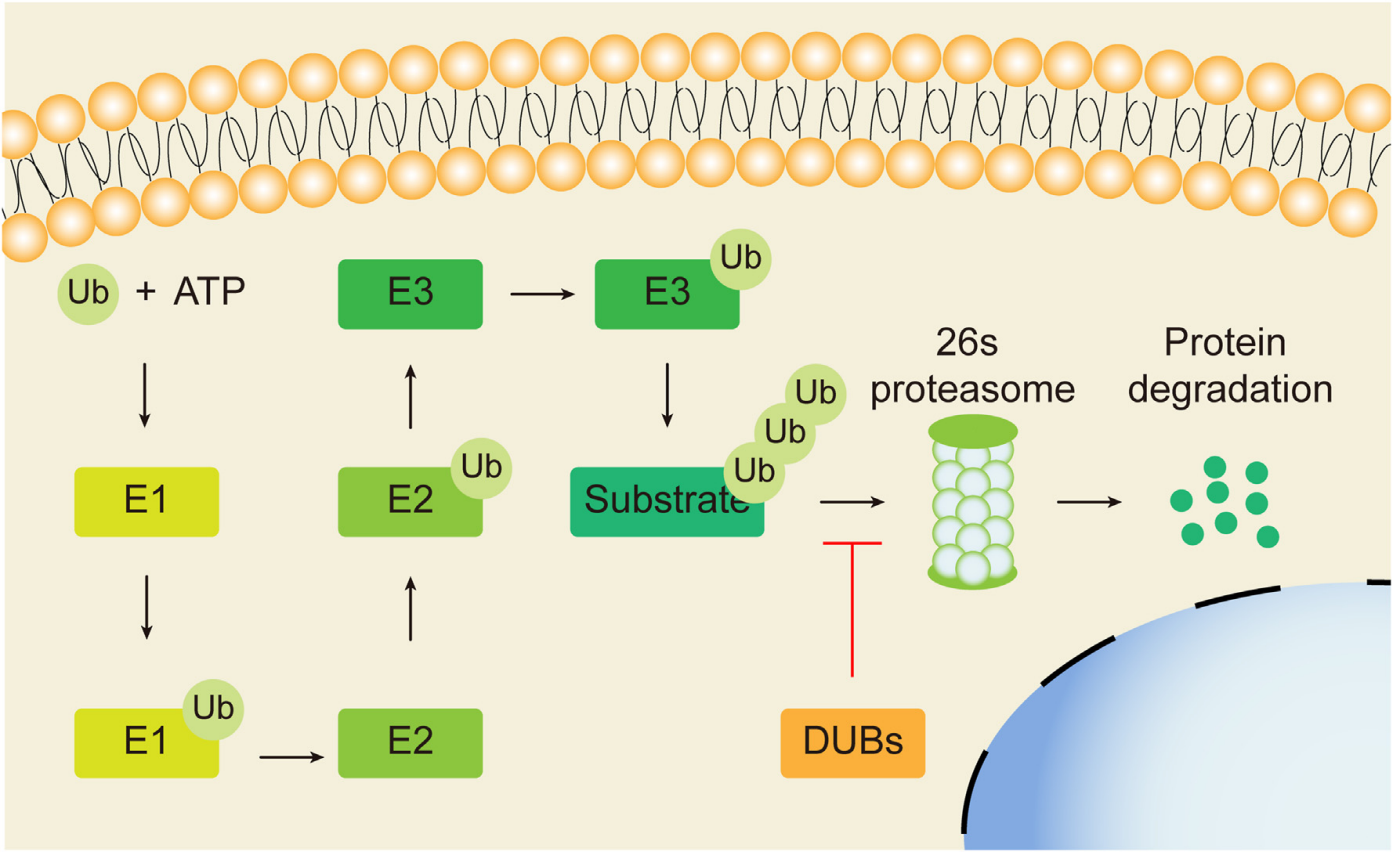
Overview of the ubiquitin-proteasome system (UPS). First, E1 activates ub after consuming ATP. Then the activated ub is transferred to the cysteine residue of E2, followed by coupling of the ubiquitin ligase E3 to the glycine at the end of ub with substrate protein or a specific lysine (K) on the ub polymerization chain. In the end, the 26S proteasome degrades the substrate protein labeled by the ub polymerization chain. The deubiquitinases (DUBs) can remove the ub chain from the substrate protein, thus reversing the ubiquitination of the substrate protein.

Ubiquitin and deubiquitination cooperate to maintain the balance of protein metabolism in eukaryotic cells.^59^ Here, E1 activates ub after consuming ATP, while the activated ub is transferred to the cysteine residue of E2, followed by coupling of the ubiquitin ligase E3 to the glycine at the end of ub with substrate protein or a specific lysine (K) on the ub polymerization chain. In the end, the 26S proteasome degrades the substrate protein labeled by the ub polymerization chain. On the contrary, the DUBs can remove the ub chain from the substrate protein, thus reversing the ubiquitination of the substrate protein (Fig. 2).^51^

### The relationship between UPS and cancer

Ubiquitination regulates many complex cellular processes, including protein degradation, protein–protein interactions, endocytosis, cell cycle progression, and the activation or inactivation of substrates.^51^ Therefore, any functional mutation or abnormal expression of UPS components can lead to certain diseases, such as tumors, neurodegenerative diseases, and adaptive or innate immune-related diseases. The physiological function of ubiquitination is not only in the hydrolyzed proteins, but also in non-hydrolyzed proteins, such as the polyprotein complex assembly, inflammatory signaling, autophagy, DNA repair, and regulation of enzyme activity.^60^, ^61^, ^62^

In tumors, ubiquitination may cause activation or inactivation of tumorigenic pathways. Abnormal expression of E3 ligase and DUBs is associated with human malignant tumors through the regulation of the activity and degradation of tumor-promoting or tumor-suppressor proteins. Typical examples include cell cycle-dependent kinase inhibitor 1B (p27), p53, transforming growth factor-beta, and noncanonical nuclear factor-kappa B.^63^, ^64^, ^65^ Unlike kinases, most components of the UPS do not have typical catalytic sites and require dynamic rearrangement of a variety of protein–protein interactions, which make it difficult for the system to be inhibited by small molecules. However, with the progress of science and technology, a deeper understanding of ubiquitin biology, and the use of the latest technologies, great progress, such as the application of PROTACs and hydrophobic tags (HyT), has been made in the reactivation of the UPS.^66^^,^^67^

### Target UPS to treat hematological malignancies

In recent years, a large number of clinical trials have been conducted on compounds targeting UPS, and some compounds have been approved for the treatment of hematological tumors. The most relevant example is the development of bortezomib, a selective inhibitor of 26S proteasome, which has become the first-line combination therapy option for multiple myeloma and mantle cell lymphoma and is also being studied in other malignant tumors.^11^^,^^68^ In addition, thalidomide derivatives, such as lenalidomide, have shown good efficacy in myelodysplastic syndrome, laying the foundation for the development of leukemia-related drugs. Existing studies are not limited to optimizing proteasome inhibitors, inhibiting a single E3 ligase or specific proteasome-related DUB enzyme, but using E1 enzyme as a target or developing a PROTAC system to stimulate ubiquitin and protein degradation has also gained great attraction.

E1 inhibitors are considered to have more extensive and effective biological effects than proteasome inhibitors because they interfere with proteasome degradation and ub-dependent signaling pathways. MLN4924 can inhibit E1 enzymes which are in charge of cancer neddylation, and phase III clinical trials (NCT04090736, NCT03268954) have been carried out in patients with leukemia.^69^ MLN7243, which belongs to a first-in-class ubiquitin-activating enzyme inhibitor, is currently conducting phase I clinical trials (NCT03816319) in patients with leukemia.

Although the extensive protein–protein interaction of E3 ligase limits the research and development of E3 ligase inhibitors, its properties that determine the specificity of ubiquitin substrates are of great attraction to researchers and many E3-based drugs are in clinical trials. Iberdomide (CC-220) is a cereblon E3 ligase regulator, which has a strong ability to degrade the IKAROS family zinc finger 1/3.^70^ Clinical trials have been carried out in B-cell lymphoma and multiple myeloma. In addition, other similar cereblon E3 ligase regulators are conducting clinical trials of related monotherapy or combination therapy: CC-12 for diffuse large B cell lymphoma, CC-90009 and BTX-1188 for acute myeloid leukemia, CFT-7455 for multiple myeloma and non-Hodgkin's lymphoma, CC-99282 for relapsed/refractory non-Hodgkin's lymphoma, and CC-92480 for relapsed/refractory multiple myeloma.

Because DUBs can inhibit the degradation of some oncoproteins, DUB inhibitors have the potential to become a cancer treatment strategy. However, due to its lack of specificity, the reported clinical trials are limited. USP11 inhibitors (mitoxantrone) (NCT02724163, NCT05313958) and USP14 inhibitors (6-mercaptopurine (NCT00866918, NCT00482833), 6-thioguanine (NCT00549848)) are currently being studied in leukemia.

Compared with the above inhibitors, proteasome inhibitors are the most successful models. At present, a variety of drugs have been published or are under development (bortexomib, carfilzomib, ixazomib, oprozomib, delanzomib, and marizomib). Bortexomib is effective in anti-multiple myeloma and mantle cell lymphoma. The efficacy of oprozomib monotherapy was confirmed in multiple myeloma.^71^ Delanzomib is highly toxic and no updated results have been reported.^72^ At present, the problems of proteasome inhibitors to be solved urgently are the emergence of drug resistance, poor pharmacokinetics, and side effects.

## The UPS in CML TKI-resistance

### E3 ligase

#### SKP2

The S-phase kinase-associated protein 2 (SKP2), also known as p45, is a key component of the SKP1-cullin1-F-box complex and is responsible for substrate recognition.^73^^,^^74^ SKP2 mainly acts as an oncoprotein, and p27 is its classic substrate protein. p27 is a kind of kinase inhibitor that is dependent on cyclin and mainly drives cells from the G1 cell division phase to the S phase. SKP2-mediated ubiquitination of p27 plays an important role in a variety of cancers, including hepatocellular carcinoma, gastric cancer, and lung cancer,^75^, ^76^, ^77^ while recent evidence additionally revealed that SKP2 is closely related to BCR-ABL-induced CML.

BCR-ABL can disrupt cell cycle regulation by disabling p27. A study found that in BCR-ABL overexpressed cell lines, p27 was negatively correlated with SKP2 expression. The inhibition of BCR-ABL would lead to the down-regulation of SKP2 and up-regulation of p27, causing cell cycle arrest. During BCR-ABL infection, the survival time of mice receiving *SKP2*^*−/−*^ bone marrow transplantation was longer than that of *SKP2*^*+/+*^ recipients, with higher nucleus expression of p27. This study confirmed that SKP2 plays an important role in BCR-ABL-induced leukemia, and inhibition of the BCR-ABL/SKP2/p27 axis may be useful in the treatment of CML patients.^78^

BCR-ABL was also found to regulate the stability of the SKP2 protein. After knocking down BCR-ABL or imatinib treatment, the SKP2 protein was down-regulated earlier than its mRNA, and the protein's half-life decreased. Further studies confirm that knocking down BCR-ABL or imatinib treatment decreased the level of Emi1, an endogenous inhibitor of E3 ligase APC/Cdh1, which mediates SKP2 degradation, thus promoting the degradation of SKP2 protein and inhibits the proliferation of CML cells.^79^

MYC is a classical oncoprotein, which is also associated with SKP2-participating CML. A correlation was previously noted between the mRNA levels of MYC and SKP2 in CML bone marrow samples. The conditional expression of MYC in K562 cells increased SKP2 mRNA and protein expression as well as decreased the p27 protein. MYC could also bind to a promoter region of the SKP2 gene and silencing SKP2 could block the regulation of p27 by MYC. These data suggest that MYC acts directly on SKP2, positively regulates SKP2 expression, and inhibits p27.^80^

In the K562 cell line, low-dose imatinib treatment induced proliferation arrest, erythroid differentiation, and up-regulated p27. A study found that MYC inhibited erythroid differentiation induced by imatinib and eliminated the up-regulation of p27. Studies that evaluated mechanisms revealed that this process partly depends on the expression of SKP2 induced by MYC. These studies additionally confirm the importance of the MYC/SKP2/p27 axis in CML progress.^81^

Cyclin-dependent kinase subunit 1 (CKS1) is the key rate-limiting component of Skp1-Cullin1-SKP2 (SKP1-cullin1-F-box (SKP2)) ubiquitin ligase, which regulates cell cycle inhibitors, connects SKP2 with p27, and promotes p27 ubiquitination. The study of V Tomiatti indicated that CKS1 expression was increased in CML, and the absence of CKS1 led to the accumulation of SKP1-cullin1-F-box (Skp2)/CKS1 substrate p21, p27, p57, and p130. These changes weakened hematopoietic stem cell proliferation, slowed regeneration after stress, and prolonged hematopoietic stem cell quiescence. Concurrently, CKS1 is also closely related to the independent cloning of cytokines induced by BCR-ABL.^82^

Bortezomib, a protease inhibitor, has been approved to be used in patients with multiple myeloma. The drug down-regulates the expression of SKP2, promotes the accumulation of p27 Kip1, and increases the apoptosis of CML cells, and therefore, is expected to become a new treatment of choice for patients with CML.^83^

In another study, Chen et al found that the high expression of SKP2 is beneficial to the proliferation of CML cells, while cyclic adenosine monophosphate response element binding protein (CREB) in the PI3K/AKT signaling pathway positively regulates the expression of SKP2, and the PI3K/AKT/CREB/SKP2 axis regulates the sensitivity of K562 cells to imatinib.^84^

Taken together, the BCR-ABL/SKP2/p27 axis is key in the occurrence and development of CML, and targeting SKP2 may show great potential in TKI-resistance CML.

#### CBL

The Casitas B-lineage lymphoma (CBL) gene encodes the E3 ubiquitin ligase and signal adaptor. The gene's repeated somatic mutations occur in myeloid neoplasms,^85^, ^86^, ^87^, ^88^, ^89^, ^90^, ^91^, ^92^ including in 10%–20% of chronic myelomonocytic leukemia patients and 10% of children with juvenile myelomonocytic leukemia, and are associated with poor prognosis.^93^, ^94^, ^95^ CBL affects the occurrence, development, and drug therapy of CML through its function of mediating substrate degradation and transferring signals.

Both TPbeta and FPalpha are chimeric oncogenes encoding constitutively active tyrosine kinase proteins found in myeloid neoplasms. Federica Toffalini's team found that in contrast to the rapid degradation of corresponding wild-type receptors after activation, the TPbeta and FPalpha hybrids were more stable and could stimulate cell proliferation more effectively. A subsequent result found that the escape of the two heterozygotes was related to the decreased degree of ubiquitination mediated by CBL.^96^

Arsenic has been used to treat acute promyelocytic leukemia and is also able to induce cell apoptosis and target BCR-ABL degradation in CML cells.^97^^,^^98^ Mao et al further confirmed that arsenic sulfide (As (4) S (4)) can promote the ubiquitination of BCR-ABL by up-regulating the expression of c-CBL. This data provides a theoretical application of arsenic combined with TKI in CML patients and treatment options for patients with TKI resistance.^99^

Moreover, CBL gene mutations can be detected in a small number of patients with chronic myelomonocytic leukemia. In order to study the role of mutant CBL in the pathogenesis of chronic myelomonocytic leukemia, Yuichiro Nakata constructed CBL conditional knock-in mice expressing chronic myelomonocytic leukemia-related CBL mutant CBL^Q367P^. The mutant has the typical characteristics of continuous proliferation of bone marrow monocytes, multilineage dysplasia, and splenomegaly (chronic myelomonocytic leukemia). The results suggest that the PI3K-AKT and JAK-STAT signaling pathways of hematopoietic stem cells in CBL^Q367P^ mice were activated, the process of the cell cycle was accelerated, and the activity of the chemokine–chemokine receptor was enhanced. Additionally, the CBL^Q367P^ mutant enhanced the activity of hematopoietic stem cells by up-regulating GEM gene expression.^100^

Belizaire et al combined functional analysis with global mass spectrometry and found that in CBL mutant cells, the interaction of activated Lck/yes-related protein tyrosine kinase (LYN) and mutant CBL drives the phosphorylation of CBL, the recruitment of PIK3R1, and the activation of the PI3K-AKT signaling pathway. Dasatinib treatment blocks these processes and inhibits the proliferation of CBL mutant cell lines and chronic myelomonocytic leukemia cells, by inhibiting LYN.^101^

Interestingly, a tyrosine kinase network composed of a TAM receptor, AXL, and the cytoplasmic enzymes, LYN and SYK, is associated with CML nilotinib resistance.^102^ Romain Gioia and colleagues found that the up-regulation of AXL and LYN in nilotinib-resistant CML cells was found to be associated with the down-regulation of CBL. Overexpression of CBL, on the other hand, reduced the expression of AXL and LYN and increased the sensitivity of cells to nilotinib. CBL was also discovered to inhibit the AXL/SYK signal by affecting the protein stability of AXL, thus promoting the transcription of LYN.^103^

The activation of JAK2 is also an important carcinogenic factor for hematological tumors. Lv et al found that CBL regulates the ubiquitination of JAK2, blocks JAK2 signal transduction, and inhibits cell growth, as well as delays the progression of invasive myeloid leukemia in mice.^104^

#### FBXW7

Leukemia-initiating cells (LIC) cannot be effectively eradicated by imatinib, which is one of the causes of disease recurrence in patients with CML. It is thus, of great clinical significance to explore the molecular mechanism of its functional regulation.

The F-box protein Fbxw7 is the substrate recognition component of the Cullin-1/SKP1-cullin1-F-box complex, which targets the ubiquitination of specific substrate proteins (classical oncoproteins such as NOTCH1, TP53, and SKP1) and is reported to play a key role in maintaining the resting state of adult hematopoietic stem cells.^105^, ^106^, ^107^, ^108^, ^109^, ^110^ c-MYC is closely related to cell proliferation and quiescence, and it has been reported that the abnormal cell cycle caused by the accumulation of c-MYC in hematopoietic stem cells and subsequent hematopoietic stem cell failure, is related to the loss of FBXW7.^111^^,^^112^ Based on the above background, Linsey Reavie and his team used CML as a LIC-dependent malignant tumor model. It was found that knocking down FBXW7 leads to the overexpression of c-MYC, inhibits the initiation of CML induced by BCR-ABL, and limits the survival and maintenance of CML LIC by activating the p53 signaling pathway. Salvage experiments show that reducing the expression of c-MYC or inhibiting the activation of the p53 signal pathway can ensure the survival and maintenance of LIC. In the same year, Shoichiro Takeishi's research showed that after deleting FBXW7, LIC was more sensitive to imatinib. The combined application of targeting FBXW7 and imatinib greatly increased the depletion of LIC. The outcome from these two studies shows the great potential of FBXW7 as an attractive target for overcoming drug-resistant CML.^113^^,^^114^

#### CHIP

Degradation of BCR-ABL is one of the effective ways to overcome CML TKI resistance. Carboxyl terminus of the Hsc70-interacting protein (CHIP) is a negative regulator of Hsc70 chaperone ATPase activity and an E3 ubiquitin ligase for the quality control of unfolded or misfolded proteins.^115^, ^116^, ^117^ Fujiko Tsukahara's study found that both E3 ubiquitin enzymes, CHIP and c-CBL, promoted the degradation of BCR-ABL protein and inhibited BCR-ABL-dependent cell growth. The former degraded immature BCR-ABL while the latter degraded mature BCR-ABL. CHIP recognizes BCR-ABL through B cell leukemia/lymphoma 2-associated athanogene-1 (BAG1), which has a high affinity for BCR-ABL. The affinity is enhanced by CHIP and heat shock protein 90 and was inhibited by imatinib and HSC70. CHIP also promotes the binding of BAG1 to BCR-ABL by inhibiting HSC70, while BAG1 promotes the binding of immature BCR-ABL to proteasome.^118^

In another study, Hu's team found that the checkpoint kinase 1 inhibitors, AZD7762 and MK-8776 had significant killing effects on drug-resistant or non-drug-resistant CML cell lines and primary cells, but conferred less damage to normal cells. Checkpoint kinase 1 inhibitors promote apoptosis of CML cells by targeting BCR-ABL, which depends on the CHIP-mediated ubiquitin degradation pathway.^119^

Another team designed a chimeric ubiquitin ligase SH2-U-box, whereby SH2 comes from the adaptor protein Grb2 and is responsible for binding activated BCR-ABL. Moreover, the U-box from CHIP acts as the E3 ubiquitin ligase domain, targeting BCR-ABL with or without T315I mutation for ubiquitination. The results suggest that the SH2-U-box can significantly promote the apoptosis of CML cells, inhibit the growth of K562-nude mouse transplanted tumor or K562R-server combined immune-deficiency mouse transplanted tumor, primary CML cells, and downstream signaling pathway of BCR-ABL.^120^ To sum up, CHIP plays an important role in targeting the BCR-ABL protein degradation pathway, and more data are needed on the molecular mechanisms of CHIP in overcoming CML resistance.

#### SIAH2

As an E3 ubiquitin ligase, seven in absentia homolog 2 (SIAH2) participates in the degradation of many proteins and affects several signal pathways, such as RAS, hippo, and hypoxia response.^121^ In CML, SIAH2 affects the efficacy of TKI by regulating hypoxia response and participating in the self-renewal of leukemic stem cells. Huang et al showed that the expression of SIAH2 in CML-blastic crisis patients was higher than that in CML-chronic phase patients, and its expression in TKI-resistant cell lines was higher than that in TKI-non-resistant cell lines under a hypoxia environment.

Vitamin K3, an inhibitor of SIAH2, can change the expression of the hypoxia-related molecules, PHD3, HIF1, and VEGF as well as increase the sensitivity of imatinib.^122^ Chun Shik Park's team found that Krüppel-like factor 4 gene deletion can increase levels of dual-specificity tyrosine-(Y)-phosphorylation-regulated kinase 2 (DYRK2). This inhibits the survival and self-renewal of CML stem cells by consuming c-Myc protein and activating the p53 signal pathway. SIAH2 can mediate the ubiquitination of DYRK2, while vitamin K3 can promote apoptosis and damage the self-renewal ability of LSCs by stabilizing DYRK2.^123^ These results suggest that SIAH2 plays an important role in overcoming the TKI resistance of CML.

#### TRAF6

Tumor necrosis factor receptor-associated factor 6 (TRAF6) is a member of the TRAF family and one of the most important E3 ubiquitin ligases. It plays an important role in a variety of tumors including lung and liver cancer and is closely related to autophagy and inflammation.^124^, ^125^, ^126^ In the past two years, studies found that TRAF6 is involved in the occurrence and development of leukemia. Tomoya Muto's team confirmed that the loss of TRAF6 in preleukemic cells is one of the causes of myeloid leukemia, and its mechanism may be related to the stem cell characteristics of the MYC oncogene. TRAF6 blocks the function of the MYC oncogene by antagonizing the acetylation of MYC.^127^

Another study found that TRAF6 is associated with TKI resistance in CML patients. Grancalcin is highly expressed in imatinib-resistant CML-chronic phase patients, and its inhibition of CML cell apoptosis is related to the activation of autophagy. The results of the mechanism-based study suggested that grancalcin induced the K63-linked polyubiquitination of TRAF6 and led to its dimerization. The TRAF6 then activates the ubiquitination of unc-51-like autophagy-activating kinase 1 (ULK1), a key downstream autophagy regulator, while the grancalcin-TRAF6-ULK1 regulatory axis regulates imatinib resistance by inducing autophagy.^128^

#### Others

The large HERC E3 ubiquitin ligase family members, HERC1 and HERC2, can interfere with a wide range of biological processes, but there are relatively few studies of their mechanisms in hematological tumors. Some studies have demonstrated a close relationship between HERC1 and BCR-ABL, which can be directly tyrosine phosphorylated by ABL kinase.^129^ In addition, the expression of HERC1 and HERC2 was negatively correlated with BCR-ABL and was time-dependent towards TKIs. The results also suggested that the differential expression of HERC1 is related to the differentiation pedigree of leukemic cells.^130^

While exploring the mechanism of drug resistance in CML, a study found that the inactivation of the cullin3 adapter gene encoding leucine zipper-like transcription regulator 1 led to increased activity of mitogen-activated protein kinase pathway and reduced sensitivity to TKIs. Further results suggest that leucine zipper-like transcription regulator 1 regulates the ubiquitination of RAS and the activation of the mitogen-activated protein kinase signaling pathway.^131^

Sun-Yong Kim and colleagues found that a non-thermal plasma-treated solution induced the death of a variety of leukemic cells (acute myeloid leukemia and CML). Mechanism-based studies revealed that non-thermal plasma-treated solution-derived reactive oxygen species kill leukemic cells by affecting the expression of E3 ubiquitin ligase ring finger protein 126 and lysosomal function to mediate the ubiquitination of mammalian target of rapamycin, which provides a new therapeutic option for chemotherapy-resistant leukemia.^132^

Yin et al found that berberine significantly inhibited the cell viability of imatinib-resistant and non-resistant cells by recruiting E3 ubiquitin ligase leucine-rich repeat and sterile alpha motif containing-1 (LRSAM1) to degrade BCR-ABL through the autophagy lysosome pathway.^133^ Tamalika Paul's research further indicated that the activation of c-Jun N-terminal kinase in mitogen-activated protein kinase signaling pathway can promote the expression of E3 ubiquitin ligase, itchy E3 ubiquitin protein ligase, enhance the binding of this E3 to FADD-like interleukin-1beta-converting enzyme-inhibitory protein and thus promote the ubiquitination of the protein. This promotion then kills imatinib-resistant leukemic cells (K562-R) by down-regulating the anti-apoptotic protein.^134^

**Figure 3:**
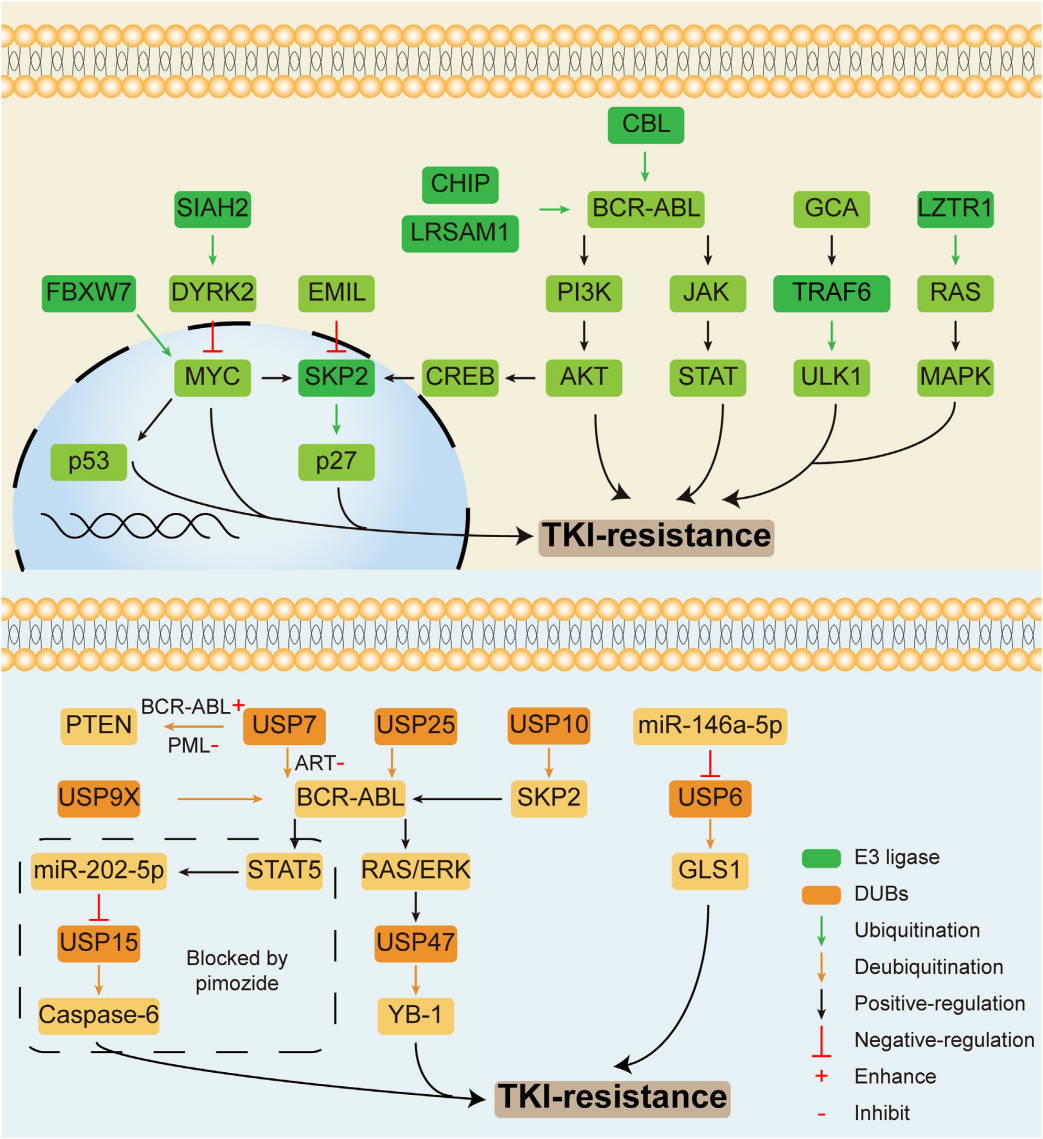
The role of E3 ligase and deubiquitinases (DUBs) in TKI resistance. For the E3 ligase, CHIP, LRSAM1, and Casitas B-lineage lymphoma (CBL) can directly target the ubiquitination of BCR-ABL and CBL can then affect TKI resistance via PI3K/AKT and JAK/STAT signaling pathway. Down-regulated FBXW7 can overcome TKI resistance by increasing the MYC protein expression and activating p53. On the contrary, low expression of SIAH2 inhibits the MYC expression by up-regulating DYRK2 to overcome TKI resistance. SKP2 can be regulated by EMIL, MYC, and PI3K/AKT/CREB axis and affect TKI resistance by negative control of p27. Besides, the GCA/TRAF6/ULK1 and LZTR1/RAS/MAPK axis can also help to confront TKI resistance. For the DUBs, USP9X, USP7, and USP25 can directly deubiquitinate BCR-ABL and USP10 can indirectly deubiquitinate BCR-ABL via SKP2. BCR-ABL can affect USP47 through RAS/ERK and STAT5 signaling pathways and further positively regulate Y-box binding protein 1 (YB-1). Also, BCR-ABL/STAT5/miR-202-5P axis can regulate USP16 and the latter can influence TKI resistance via caspase-6. Moreover, the miR-146a-5p/USP6/GLS1 axis can help to overcome TKI resistance without BCR-ABL involvement. TKI, tyrosine kinase inhibitor; ULK1, unc-51-like autophagy-activating kinase 1; GCA, grancalcin; LZTR1, leucine zipper-like transcription regulator 1; MAPK, mitogen-activated protein kinase; ERK, extracellular signal-regulated kinase; GLS1, glutaminase 1.

Based on the above studies, an in-depth understanding of E3 ligases will help to explore the mechanism of CML and drug resistance and provide a new treatment strategy for CML drug-resistant patients (Fig. 3 and Table 1).

**Table 1:** The mechanisms of how the ubiquitin-proteasome system is involved in tyrosine kinase inhibitor resistance.

Name	Role in the ubiquitin-proteasome system	Involvement in tyrosine kinase inhibitor resistance	Associated signaling pathways or molecules
SKP2	E3 ligase	Target the downstream signaling pathway of CML	BCR-ABL/MYC/SKP2/p27 axis and PI3K/AKT/CREB/SKP2 axis
CBL	E3 ligase	Directly target the ubiquitination of BCR-ABL	PI3K-AKT and JAK-STAT signaling pathway
FBXW7	E3 ligase	Target leukemia-initiating cells (LIC)	c-MYC and p53
CHIP	E3 ligase	Directly target the ubiquitination of BCR-ABL and target leukemic stem cells (LSCs)	
SIAH2	E3 ligase	Target the downstream signaling pathway of CML and LSCs	SIAH2/DYRK2/(c-Myc or p53)
TRAF6	E3 ligase	Target the downstream signaling pathway of CML	GCA/TRAF6/ULK1 axis
LZTR1	E3 ligase	Target the downstream signaling pathway of CML	MAPK signaling pathway
LRSAM1	E3 ligase	Directly target the ubiquitination of BCR-ABL	
USP6	DUBs	Target the downstream signaling pathway of CML	miR-146a-5p/USP6/GLS1 axis
USP7	DUBs	Directly target the deubiquitination of BCR-ABL and the downstream signaling pathway of CML	BCR-ABL/USP7/PTEN axis and USP7/BCR-ABL/(STAT5 or LYN) axis
USP9X	DUBs	Directly target the deubiquitination of BCR-ABL	
USP10	DUBs	Indirectly target the ubiquitination of BCR-ABL	USP10/SKP2/BCR-ABL axis
USP14	DUBs	Directly target the deubiquitination of BCR-ABL	
USP15	DUBs	Target the downstream signaling pathway of CML	STAT5A/miR-202-5p/USP15/caspase-6 axis
USP25	DUBs	Directly target the deubiquitination of BCR-ABL	USP25/BCR-ABL/STAT5
USP47	DUBs	Target the downstream signaling pathway of CML and protect the LSCs	BCR-ABL-(RAS/REK or STAT5)-USP47-YB-1 axis
UBE1	E1 enzyme	Target the downstream signaling pathway of CML	p53 and p27
Bortezomib	Proteasome inhibitors	Inhibit CML growth and promote cell apoptosis	NF-κB2, RB, and caspase-3/8/9
Carfilzomib	Proteasome inhibitors	Promote cell apoptosis	ERK/ABI-1/2
Oprozomib	Proteasome inhibitors	Promote cell apoptosis	Caspase-12 and ASK-JNK-BIM axis
NAE1	NEDD8-activating enzyme	Target the downstream signaling pathway of CML and LSCs	p27

Notes: NF-κB2, noncanonical nuclear factor-kappa B subunit 2; ULK1, unc-51-like autophagy-activating kinase 1; GCA, grancalcin; LZTR1, leucine zipper-like transcription regulator 1; MAPK, mitogen-activated protein kinase; ERK, extracellular signal-regulated kinase; LRSAM1, leucine-rich repeat and sterile alpha motif containing-1; JNK, c-Jun N-terminal kinase; GLS1, glutaminase 1; PTEN, phosphatase and tensin homolog; YB-1, Y-box binding protein 1; ABI-1/2, Abelson interacting protein 1 and 2; NAE1, NEDD8 activating enzyme E1 subunit 1.

### DUBs

#### USP6

MicroRNAs in exosomes that are derived from mesenchymal stem cells are involved in the treatment of many diseases. Studies have shown that a large amount of miR-146a-5p in human umbilical cord mesenchymal stem cell-derived exosomes is associated with ischemic stroke, ovarian cancer, and acute leukemia.^135^ Wen's team confirmed that miR-146a-5p plays an important role in the treatment of TKI resistance in CML patients by regulating the expression of USP6. USP6 is highly expressed in bone marrow samples of patients with CML and is associated with poor prognosis. Compared with imatinib-sensitive patients, USP6 was significantly up-regulated in TKI-resistant patients. MiR-146a-5p derived from human umbilical cord mesenchymal stem cells can inhibit the high expression of USP6, leading to the enhancement of the ubiquitination of glutaminase 1, and the USP6 substrate, followed finally by the promotion of apoptosis of K562-R cells induced by imatinib mesylate. Therefore, the miR-146a-5p/USP6/glutaminase 1 signal may be a new therapeutic target for further investigation for CML patients with TKI resistance.^136^

#### USP7

USP7 plays an increasingly important role in hematological malignant tumors, especially CML.^137^^,^^138^ Pandolfi et al reported that USP7 can mediate the de-ubiquitination of tumor suppressor phosphatase and tensin homolog and enhance its nuclear exclusion. BCR-ABL enhances the preceding process by interacting with and phosphorylating USP7 through amino acid residues on USP7. In contrast, the promyelocytic leukemia gene can inhibit the de-ubiquitination of phosphatase and tensin homolog by USP7. Therefore, the reverse regulation mechanism of BCR-ABL/promyelocytic leukemia gene on USP7-mediated de-ubiquitination of phosphatase and tensin homolog may be a novel treatment of CML.^139^

Moreover, through the screening of DUBS, Mao's team found that USP7 prevented the polyubiquitin and degradation of BCR-ABL, as well as enhanced the phosphorylation of its downstream molecules, such as STAT5 and LYN. The antimalarial artesunate also promoted the degradation of BCR-ABL and induced apoptosis of CML cells by inhibiting the interaction between USP7 and BCR-ABL, making it a possible useful target for CML therapy.^140^

#### USP9X

USP9X is highly expressed in many kinds of tumors and plays the role of oncogenes. However, a few studies have suggested that USP9X has roles in hematological tumors.^141^^,^^142^ USP9X was found to inhibit cell death by inhibiting the ubiquitin degradation of myeloid cell leukemia-1 thus promoting disease progression. Myeloid cell leukemia-1 was expressed in abnormally high levels in CML, which is related to chemotherapy resistance and disease recurrence.^143^ WP1130, an inhibitor of the deubiquitin enzyme USP9X, enhanced the ubiquitin degradation of BCR-ABL, resulting in its accumulation in aggresomes, and down-regulated myeloid cell leukemia-1 to promote the apoptosis of imatinib-sensitive and drug-resistant CML cells. These results further suggest that USP9X is a promising new treatment option for CML.^144^

#### USP10

USP10 functions in cancers by regulating different substrates (androgen receptor, p53, SIRT6, AMPK, and Flt3).^145^, ^146^, ^147^, ^148^, ^149^, ^150^ Liu et al found that primary CML cells overexpressed USP10 and SKP2. USP10 mediates the de-ubiquitination and stability of SKP2, while the latter can positively modulate the expression of BCR-ABL by regulating its K63-associated ubiquitination and activation. Furthermore, the inhibition of USP10 can inhibit the growth of imatinib-sensitive and -resistant CML cells, primary CML cells, and transplanted tumors in nude mice, which process depends on the functional state of SKP2. Therefore, USP10/SKP2/BCR-ABL signal axis plays an important role in the occurrence and development of CML and may be another promising new therapeutic target for patients with TKI resistance.^151^

#### USP14/UCHL15

Based on the key regulatory role of DUBs in cellular protein degradation, DUB inhibitors are considered attractive anticancer agents. Nickel pyrithione (NiPT) is an effective inhibitor of the 19S proteasome-related deubiquitinases (UCHL5 and USP14) but does not affect the 20S proteasome.^152^ Liu et al showed that NiPT induced apoptosis of BCR-ABL-WT and BCR-ABL-T315I cells and inhibited the growth of xenografts derived from these two cells in nude mice. NiPT reduces the protein expression level of BCR-ABL by inhibiting BCR-ABL transcription and caspase-dependent cleavage. NiPT-induced UPS inhibition enhanced the activation of caspase in imatinib mesylate-resistant and -sensitive CML cells, while the activation of the latter leads to NiPT-induced down-regulation of BCR-ABL and CML cell death. These findings prove that NiPT can overcome imatinib mesylate resistance and has a high clinical transformation value.^153^ Shi et al found that the expression of 20S proteasome polypeptidase and 19S proteasome deubiquitinase (USP14 and UCHL5) in primary cells of CML patients was higher than that of healthy people. B-AP15, an inhibitor of USP14 and UCHL5, can cooperate with imatinib to inhibit the growth of BCR-ABL-WT and BCR-ABL-T315I CML cell lines, CML xenograft tumor, and primary CML cells.^154^ Meanwhile, in another study, they found that piperlongumine, isolated from piper longum L., can target USP14 and UCHL5 to inhibit the proteasome function of CML cells with or without T315I mutation, weaken the cell viability of CML cell line, induce apoptosis, and inhibit the growth of transplanted tumor. This study provides a theoretical basis for the application of piperlongumine in patients with CML resistance.^155^

#### USP15

A large number of studies have shown that STAT5 plays a key role in the pathogenesis of CML, but its downstream target genes need further exploration.^156^, ^157^, ^158^, ^159^ Previously, STAT5A affected the expression of deubiquitylases USP15 by regulating microRNA, and mediated apoptosis and imatinib resistance of CML cells. STAT5A and miR-202-5p were also found to be highly expressed in CML patients, while USP15 was minimally expressed. STAT5A activates the transcription of miR-202-5p by binding to the promoter of miR-202 and up-regulates miR-202-5p, while the latter down-regulates the expression of USP15 by targeting its 3′-UTR. Deletion of USP15 leads to the increased ubiquitination of caspase-6 and thus decreases its protein expression, which in turn inhibits CML cell apoptosis and promotes TKI resistance. More importantly, the STAT5A/miR-202-5p/USP15/caspase-6 signal axis can be blocked by pimozide, promoting the apoptosis of CML cells *in vivo* and *in vitro*. Therefore, targeting this signal axis may become a new therapeutic strategy for TKI-resistant CML patients.^160^

#### USP25

The degradation of fusion gene products depends on the quality control mechanism of proteins, while the high expression of BCR-ABL in CML cells indicates impairment of its normal degradation. Thorough research on the anti-degradation of BCR-ABL found that the deubiquitylases, USP25, inhibited the ubiquitination of BCR-ABL, thus increasing its protein level. Additionally, the deletion of USP25 induced the degradation of BCR-ABL and inhibited its downstream STAT5-related signal transduction, as well as the proliferation of Ph-positive leukemic cells. During these processes, the effect of USP25 inhibitors was not affected by TKI, making USP25 an additional target for patients with TKI resistance.^161^

#### USP47

Studies have confirmed that USP47 plays an important role in cancers such as gastric cancer, medulloblastoma, and colorectal cancer, but its functions in CML are yet to be established.^162^, ^163^, ^164^ Wu et al reported that USP47 is overexpressed in CML, and its knockdown or using the USP47 inhibitor P22077 inhibited the proliferation of imatinib-sensitive or -resistant CML cells and reduced the weight of the transplanted tumor. The knockout of USP47 or using P22077 also significantly impaired BCR-ABL and BCR-ABL^T315I^-induced CML in mice and reduced the number of CML stem/progenitor cells. In-depth mechanism studies have shown that BCR-ABL up-regulates the expression of USP47 through RAS/extracellular signal-regulated kinase and STAT5 signaling pathways. USP47 also binds to downstream Y-box binding protein 1 and protects it from proteasome degradation, making it conducive for DNA damage repair to occur in CML cells. In conclusion, USP47 is a promising target to overcome TKI resistance and eradicate leukemic stem cells/progenitor cells in CML.^165^

### E1 and E2

The specific substrate degradation function of E3 ubiquitin ligase is indifferent from the role of E1 ubiquitin-activating enzyme and E2 ubiquitin-conjugating enzyme. Although at present, the data on E3 ubiquitin ligase are much more than that of E1 and E2 enzymes, the latter two are still crucial in CML. It may be effective in inhibiting the E1 and E2 enzymes to block the increased ubiquitin in leukemia.

PYR-4 and NSC624206 are two compounds with the ability to inhibit the E1 ubiquitin-activating enzyme UBE1 by blocking the formation of ubiquitin-thioester bond, and then inhibit the ubiquitination of p53 and p27.^166^^,^^167^ The transcriptional activities and related signaling pathways of p53 and p27 play an important role in the occurrence, development, and maintenance of leukemic stem cells.^168^^,^^169^

A research team identified recurrent mutations of ubiquitin-conjugating enzyme E2A for the first time, through large-scale parallel sequencing analysis of 10 blast crisis samples and corresponding autologous chronic phase controls. The mutations were specific through further analysis and *in vitro* studies, with an incidence of 16.7% in progression, and were associated with myeloid differentiation disorders in CML.^170^

The above evidence suggests that targeting E1 and E2 enzymes will be a potential therapeutic strategy for CML but warrants further evidence.

## Proteasome inhibitors

At present, research has confirmed that the combination of TKIs and proteasome inhibitors can effectively synergize. Iris et al found that compared with using TKI alone, treating BCR-ABL plus acute lymphocytic leukemia with proteasome inhibitors bortezomib and ixazomib alone or in combination with TKI was more effective in reducing the viability of leukemia cell lines. The specific data are as follows: the cell viability after bortezomib treatment is 1.26% ± 0.46%, the cell viability after bortezomib combined with TKI treatment is 1.57% ± 0.7%, and the cell viability after dasatinib treatment alone is 40.58% ± 2.6%, which proves that TKI combined with proteasome inhibitor treatment has a great killing effect on BCR-ABL plus leukemia cells.^171^ In CML, proteasome inhibitors can attack resistant or non-resistant CML cells and promote cell apoptosis. At the same time, many studies have also confirmed that the combination of proteasome inhibitors and TKI treatment has a more significant killing effect on CML cells, proving that proteasome inhibitors have the potential to change the treatment status of patients.

### Bortezomib

Bortezomib, an inhibitor of 20S proteasome, is responsible for the degradation of a variety of intracellular proteins and shows significant activity in patients with multiple myeloma.^172^^,^^173^ Recent studies have shown that bortezomib also has a certain tumor-toxic effect on CML. Roger et al showed that bortezomib can inhibit the growth of BCR-ABL-expressing cells and promote apoptosis by inactivating noncanonical nuclear factor-kappa B subunit 2 and reducing the phosphorylation of RB. At the same time, bortezomib can induce cell cycle arrest and apoptosis of imatinib-resistant CML cells.^174^ Holyoake et al found that bortezomib can target primitive CML cells and induce CD34^+^ CML cell apoptosis at a clinically achievable concentration. Bortezomib alone or in combination with dasatinib can kill CML cells. For cell lines with BCR-ABL mutation, bortezomib can also effectively inhibit their proteasome activity and induce apoptosis.^175^ Wang's team studied the effect of bortezomib in combination with sphingosine kinase 1 inhibitor SKI in CML cell lines. The results suggest that the combination of two drugs can increase the caspase-3 cleavage and apoptosis of drug-resistant or non-drug-resistant CML cells. Mechanism studies have found that increased apoptosis is related to the down-regulation of BCR-ABL and myeloid cell leukemia-1 and the significant decrease of sphingosine kinase 1 expression.^176^ Another team's results focused on a combination of bortezomib and mitotic inhibitors, such as microtubule stabilizer paclitaxel and PLK1 inhibitor BI2536. Combination therapy showed a strong killing effect in TKI-sensitive or insensitive CML cells, effectively activating caspase-3, -8, and -9, and inhibiting the downstream signal pathway of BCR-ABL.^177^ Although the effect of bortezomib is not selective and may lead to side effects such as bone marrow transplantation, the current experimental results still suggest that bortezomib may become another treatment option for patients with CML.

### Carfilzomib

As a new generation of proteasome inhibitors, carfilzomib is an epoxyketone-based inhibitor that irreversibly binds to the proteasome. Currently, carfilzomib has been approved by the U.S. Food and Drug Administration for the treatment of recurrent/refractory multiple myeloma, showing better efficacy and fewer side effects than bortezomib.^178^^,^^179^ Based on the effective killing effect of bortezomib on CML cells, Irvine et al studied the cytotoxicity of carfilzomib in CML. The results suggest that carfilzomib inhibits extracellular signal-regulated kinase signaling and promotes the expression of Abelson interacting protein 1/2. It has a synergistic effect with imatinib and nilotinib and can effectively promote the apoptosis of primitive CML stem cells and imatinib-resistant CML cells.^180^ The results of this study provide basic support for the use of carfilzomib in the treatment of CML patients.

### Oprozomib

Oprozomib is a tripeptide analogue of carfilzomib, which is an oral bioavailable proteasome inhibitor.^181^ In the preclinical model, oprozomib showed the same anti-tumor activity as carfilzomib. In patients with recurrent/refractory multiple myeloma, oprozomib showed good efficacy as a single drug or in combination with other drugs.^182^^,^^183^ Wang's team conducted multidrug screening of bone marrow mononuclear cells from patients with CML and found that oprozomib was a candidate drug that could induce apoptosis in leukemic cells. In-depth mechanism studies have found that oprozomib can promote the up-regulation of endoplasmic reticulum chaperone protein, release calcium ions into the cytoplasm, activate calpain, and then cleave caspase-12. At the same time, it can also activate IRE1α phosphorylation and promote CML cell apoptosis through the ASK/c-Jun N-terminal kinase/BIM axis.^184^ This study provides theoretical evidence for the clinical transformation of oprozomib to CML therapy.

## Ubiquitin-like proteins

### NEDD8

Protein neddylation is a post-translational modification similar to ubiquitination, which adds a ubiquitin-like neural precursor cell expressed, developmentally down-regulated 8 (NEDD8) to the target protein. Neddylation involves three enzymes: NEDD8-activating enzyme (NAE1), E2-conjugating enzyme (UBC12), and E3 ligases.^185^ A large number of studies have shown that neddylation plays an important role in DNA damage, tumorigenesis, and drug resistance.^186^, ^187^, ^188^

NAE1 inhibitor-MLN4924 has significant cytotoxic effects against several tumors, induces DNA damage in Ph^+^ leukemia, and enhances its sensitivity to ABL kinase inhibitors. Clinical trials have been carried out in hematological malignant tumors such as acute myeloid leukemia, acute lymphocytic leukemia, and diffuse large B-cell lymphoma.^69^^,^^189^ In a study involving the overexpression of NAE1 in CML cells, the NAE1 inhibitor MLN4924 could inhibit the growth of CML cell lines and CML patient-derived CD34^+^ cells, induce cell cycle arrest and apoptosis in CML cells containing wild-type p53, regardless of their sensitivity to TKIs, and prolong the survival of CML mice and weaken the maintenance of leukemic stem cells. Further studies have found that MLN4924 can promote the nuclear accumulation of p27^kip1^ in CML cell lines and primary cells while silencing the p27^kip1^ weakens the anti-CML effect of MLN4924. Based on the above evidence, although more long-term research is needed, targeting neddylation may be a new therapeutic strategy for drug resistance in CML.^168^

### Other ubiquitin-like proteins

At present, there are no relevant research reports on the involvement of other ubiquitin-like proteins in CML resistance. However, there is considerable evidence to suggest that some ubiquitin-like proteins play an important role in CML. Interferon-stimulated gene 15 is a ubiquitin-like modifier, and its expression and binding to other proteins (ISGylation) are strongly increased under the stimulation of type 1 interferon (IFN).^190^ UBP43 is an interferon-stimulated gene 15-specific isopeptidase whose expression is activated by IFN. Studies have shown that UBP43 deficiency increases resistance to BCR-ABL carcinogenic transformation, and this resistance to leukemia development depends on the type 1 IFN (IFN α/β) signaling in UBP43 deficient cells.^191^

### Development of PROTAC in CML

As discussed, various components of the UPS either resist or promote TKI resistance of CML in multiple ways. They include directly or indirectly targeting the ubiquitination process of related molecules, inhibiting or activating CML-related downstream signaling pathways, and affecting the functional status of leukemia stem cells. Therefore, many molecules have the potential to become new targets for CML therapy in the future, and their combination with TKI is expected to be highly efficacious. However, the clinical focus on TKI resistance is still to directly target BCR-ABL, leukemia “promoter”, and constantly improvise TKI. At present, the third-generation TKI drug olverembatinib has shown good efficacy in CML patients with or without T351I mutation, making it superior to nilotinib and dasatinib.^192^

**Figure 4:**
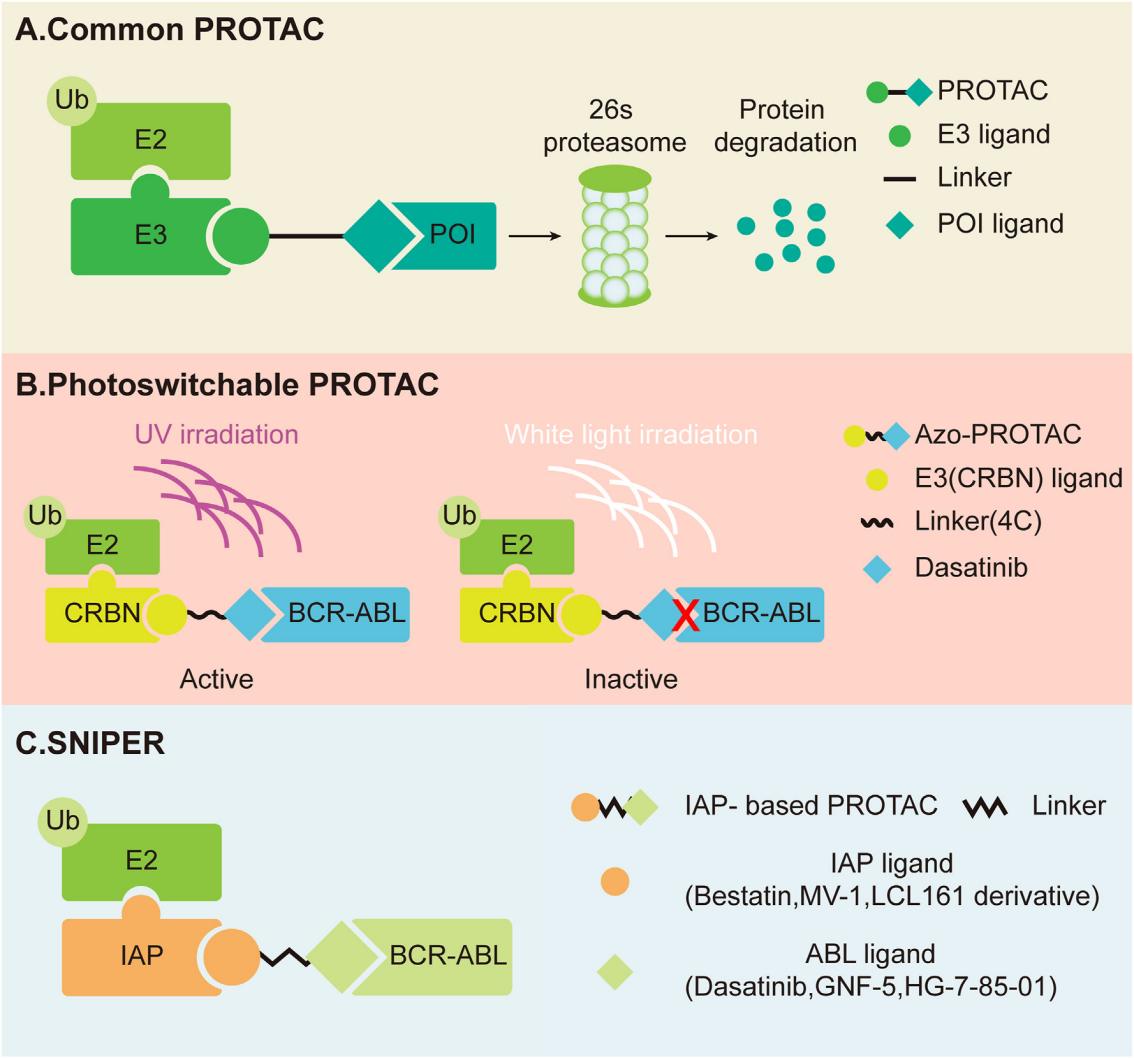
Overview of the common PROTAC and PROTAC targeting BCR-ABL. **(A)** The common PROTAC contains three parts, a ligand used to bind the POI (POI ligand), another ligand to recognize E3 ligase (E3 ligand), and a linker that connects the two ligands. PROTAC recruits both the E3 ligase and POI to form an “E3-PROTAC-POI” ternary complex. **(B)** The photoswitchable PROTAC is based on the CRBN E3 ligand and 4C (linker). Being light-controlled, the trans-4C degrades BCR-ABL rapidly at 100 nM, while cis-4C loses its degradation function at 500 nm. UV irradiation for 1 h transformed trans-4C to cis-4C, which would otherwise take 4 h with white light irradiation. **(C)** SNIPER (IAP-based PROTACs) couple ABL inhibitors (imatinib, GNF-5, HG-7-85-01, dashatinib) with IAP ligands (MEBS, MV-1, LCL161 derivatives) and can achieve the degradation of BCR-ABL. PROTAC, proteolysis targeting chimeras; POI, protein of interest.

As a classic CML therapeutic drug, the principle of TKI is to inhibit the activity of tyrosine kinase and act as a BCR-ABL inhibitor. The better aim would be to design a drug to directly target the degradation of tyrosine kinase to eliminate the leukemia “promoter” BCR-ABL. Inspired by UPS, researchers designed the proteolysis targeting chimeras (PROTAC) to use UPS to degrade proteins of interest (POI). The PROTAC molecule consists of three covalent bonds: a ligand used to bind the POI (POI ligand), another ligand to recognize E3 ligase (E3 ligand), and a linker that connects the two ligands. PROTAC recruits both the E3 ligase and POI to form an “E3-PROTAC-POI” ternary complex. A number of studies have investigated PROTAC on BCR-ABL (Fig. 4 and Table 2).^66^^,^^193^^,^^194^

**Table 2:** The basic composition of PROTAC target BCR-ABL.

Name	E3 ligand	POI ligand	Reference
The first PROTAC target BCR-ABL	CRBN	Bosutinib or dasatinib	195
GMB-475	VHL	GNF-5	196
GMB-805	VHL	ABL001	197
Azo-PROTACs	CRBN	Dasatinib	198
SIAIS178	VHL	Dasatinib	200
P19P	CRBN	Ponatinib	199
P19AS	CRBN	Asciminib	199
SNIPER (ABL)-39	LCL161 derivative	Dasatinib	201
SNIPER (ABL)-62	IAP	ABL001 derivative	202
DAS-IAP	LCL161 derivative	Dasatinib	203

Note: POI, protein of interest.

In 2016, Crew et al first designed the PROTAC that targeted BCR-ABL. The tested PROTACs used four different linkers to connect the BCR-ABL TKIs (imatinib, bosutinib, and dasatinib). The TKIs were bound to the c-ABL kinase domain to the known Von Hippel Lindau (VHL) E3 ubiquitin ligand or Cereblon (CRBN) E3 ubiquitin ligand. Among these targets, only bosutinib-CRBN and bosutinib-CRBN can degrade BCR-ABL, and the latter has stronger leukemia cell selectivity. These results show that warhead and E3 ligase are very important for the design of PROTAC targeting BCR-ABL.^195^

In 2019, Crew's team used structurally modified GNF-5 to combine with VHL binding elements, taking advantage of the former's function of improving cell permeability and affinity, to optimize the structure of PROTAC and synthesize GMB-475. GMB-475 mediates the degradation of wild-type and mutant BCR-ABL (T315I and G250E) and enhances the sensitivity of TKI-resistant cells to imatinib. GMB-475 also promoted the apoptosis of CML CD34^+^ cells without affecting normal CD34^+^ cells.^196^ Next, Crew found that there was a complementary synergistic effect by combining imatinib and GMB-475, which could significantly reduce TKI resistance, drug toxicity, and adverse reactions.

In a follow-up study, a scaffold hopping approach was applied to enhance the activity of the GMB-475 and obtain a new PROTAC (GMB-805). As GMB-805 eliminated the need to optimize linkers to access more efficient degraders, its degradation induction ability was over 10 times higher than that of GMB-475 and demonstrated strong anti-leukemia cell proliferation abilities. The above studies reveal that PROTAC targeting BCR-ABL has great prospects as a therapeutic for CML patients and CML TKI-resistant patients when combined with TKI and PROTAC in the future.^197^

In addition to Crew's research, there are also other scientists concerned about BCR-ABL PROTAC. Jiang and his colleagues designed a light-controlled photoswitchable azobenzene-proteolysis targeting chimeras (Azo-PROTACs). Among many Azo-PROTACs, the Azo-PROTAC-4C based on the CRBN E3 ligand and 4C (linker) exhibited the best activity in degrading the BCR-ABL fusion protein. Being light-controlled, the trans-4C degrades BCR-ABL rapidly at 100 nM, while cis-4C loses its degradation function at 500 nm. UV irradiation for 1 h transformed trans-4C to cis-4C, which would otherwise take 4 h with white light irradiation. Azo-PROTAC not only targets BCR-ABL degradation but also achieves artificial control, creating broader prospects with the advancement of precision medicine.^198^ Rao and the team further paired ponatinib and asciminib with CRBN to produce BCR-ABL PROTAC (P19P and P19AS). Both combinations lowered the level of BCR-ABL in K562 cells and BaF3 cells carrying the T315I mutation. Moreover, P19P had less cytotoxicity compared with ponatinib.^199^ In another report, dasatinib was paired with VHL-1, thus designing a new PROTAC (SIAIS178). The results suggest that SIAIS178 can degrade wild-type BCR-ABL and various mutants of BCR-ABL (T315I, F317V, F317L, G250E, and V299L), and significantly inhibit the growth of K562 cells *in vitro* and *in vivo*.^200^

In addition to VHL and CRBN, the E3 ubiquitin enzyme IAP was also designed as PROTAC to target BCR-ABL degradation. It is called the specific and non-genetic inhibitors of apoptosis protein (IAP)-dependent protein erasers (SNIPER). In 2017, Naito designed a series of IAP-based PROTACs by coupling ABL inhibitors (imatinib, GNF-5, HG-7-85-01, dashatinib) with IAP ligands (MEBS, MV-1, LCL161 derivatives). Among these combinations, SNIPER (ABL)-39 effectively degraded BCR-ABL, inhibited its downstream signaling pathway, and damaged the proliferation of CML cells.^201^ Subsequently, Naito designed SNIPER (ABL)-62 and DAS-IAP, which were better than SNIPER (ABL)-39 in all aspects.^202^^,^^203^ Based on these data, IAP-based PROTACs demonstrate a greater development potential as a treatment.

At present, there is no clinical trial of PROTAC in the treatment of CML, but in other tumors, PROTAC has shown good characteristics in preclinical and clinical settings.^204^ Some problems and obstacles need to be addressed before PROTAC can be used more widely in the treatment of patients. Although the data received so far are limited, similar to TKI, a small number of tumor patients are resistant to protein degradants. It has been found that shared and drug-specific modulator networks, including the constitutive photomorphogenesis 9 signalosome and the substrate receptors exchange factor CAND1 can induce resistance to protein degradation agents by affecting the activity of E3 ubiquitin ligase.^205^ Another study has shown that the loss of related ubiquitin proteasome system components can cause cancer cells to become resistant to degraders.^206^ In addition, Shen et al found that acquired resistance to VHL and CRBN-based BET-PROTAC is mainly due to genome changes that damage the core components of the related E3 ligase complex.^207^ To sum up, the existing evidence suggests that PROTAC-related drug resistance is mainly due to changes in UPS, and target protein-related drug resistance has not been reported. However, due to the limited published basic research and the lack of long-term follow-up data for patients treated with protein degradants, the current conclusions may not be accurate, and more experimental and clinical data are needed to explore the mechanism of PROTAC resistance in the future.

## Conclusions and prospects

The advent of TKI targeting BCR-ABL has greatly changed the treatment of CML and prolonged the survival and quality of life of patients with CML. The research and development of TKI are ongoing and the new generation of TKIs has a wider range of indications. These TKIs induce deeper and faster remission and have fewer adverse reactions, but drug resistance is still a primary problem seriously affecting the prognosis of CML patients. In addition to developing novel BCR-ABL inhibitors, new targets are being explored. In view of the important biological functions of UPS in the human body, it appears as a key agent to help overcome CML drug resistance. The components of UPS can be roughly divided into two categories: “protein degraders” (E1, E2, and E3 ubiquitinases) and “protein degradation antagonizors” (DUBs and proteasome inhibitors). Although mediating opposite physiological activities, their roles in CML TKI resistance are diverse and not directly antagonistic due to targeting different substrates or signaling pathways. Firstly, they directly or indirectly target the degradation of BCR-ABL (CHIP, LRSAM1, c-CBL, SKP2, USP9X, USP7, USP10, USP25) and can combine with TKIs to help treat CML. Secondly, they activate or inhibit the downstream signaling pathway of CML (FBXW7, TRAF6, leucine zipper-like transcription regulator 1, c-CBL, USP47, USP6, USP15, bortezomib, carfilzomib, oprozomib), so that they can help to eliminate CML cells which evade the killing effect of TKIs. Finally, they affect the state of leukemia stem cells (CHIP, SIAH2, bortezomib, carfilzomib) and avoid recurrence and drug resistance of CML caused by LSCs. The presence of LSCs is one of the main reasons for TKI resistance in CML patients and has been a research hotspot in recent years. At present, many studies have confirmed that many signaling pathways can regulate CML LSCs, but their specific regulatory mechanisms have not been fully elucidated. Because there is limited research about UPS involved in CML LSCs, there is still great room for exploring the role of UPS in them. Except for the traditional UPS components, PROTAC technology has become a novel strategy for new drug research and development. PROTAC, a protein degrader targeting BCR-ABL, is presumed to surpass the protein inhibitor TKI. So far, 18 preclinical studies related to PROTAC in the treatment of cancer are underway, of which 7 are related to hematological malignancies. The seven clinical trials are all in phase I; five of them target Bruton tyrosine kinase and concentrate on B cell malignancies; one focuses on B-cell non-Hodgkin's lymphoma, targeting interleukin 1 receptor associated kinase 4; and the other one focuses on eligible blood cancers, targeting signal transducer and activator of transcription 3 (Table 3).^204^ Although there is no clinical and preclinical data on PROTAC in the treatment of CML, it is undeniable that this technology will develop in the next few years, similar to the CAR-T cell technology, be fully explored for applications in TKI-resistant CML patients, and change the existing strategy of CML treatment. The innate immune response plays a key role in inhibiting the development of cancer, and the activation of the interferon type 1 (IFN-α/β) signal pathway is a key component of it.^208^, ^209^, ^210^ Before the advent of TKI, IFN-α was the standard treatment choice for patients with CML who did not meet the criteria for bone marrow transplantation. However, due to many side effects and a low initial reaction rate, IFN-α was gradually replaced by TKI. In recent years, the problem of drug resistance of TKI has been perplexing some CML patients, but some studies have found that after stopping the administration of IFN, there are still a considerable number of patients with IFN responses who can be completely relieved.^211^^,^^212^ At the same time, research has found that the killing effect of IFN on leukemic stem cells is stronger than that of imatinib.^213^ Therefore, it is very important to explore the molecular mechanism of the clinical response of IFN and clarify the role of immune response in the treatment of CML. In the above text, we described that IFN can enhance the endogenous IFN response by inhibiting Ubp43 or other negative regulators of IFN signal, and can be used in combination with imatinib as a useful drug for the treatment of BCR-ABL-induced leukemia.^191^ This study shows that UPS can participate in the occurrence and development of CML and drug resistance of TKI by affecting immune response. Although the evidence is limited, it is also a potential research direction. Moreover, the continuous development of TKIs revealed promising results, while more studies confirm the many components of UPS as CML therapeutic targets. This paper briefly describes the mechanism of TKI drug resistance, describes the basic composition of UPS and the process of protein ubiquitination, and focuses on the research progress of UPS-related molecules on CML TKI resistance, in order to provide reference for the rational development and design of new CML therapeutic targets.

**Table 3:** Clinical trials of PROTAC in hematological malignancies.

PROTAC	E3 ligand	Target	Disease	NCT number
NX-2127	CRBN	BTK	B cell malignancies	NCT04830137
NX-5948	CRBN	BTK	B cell malignancies	NCT05131022
BGB-16673	CRBN	BTK	B cell malignancies	NCT05006716
BGB-16673	CRBN	BTK	B cell malignancies	NCT05294731
HSK29116	CRBN	BTK	B cell malignancies	NCT04861779
KT-413		IRAK4	B cell non-Hodgkin lymphoma	NCT05233033
KT-333	CRBN	STAT3	Blood cancers and solid tumors	NCT05225584

Notes: STAT3, signal transducer and activator of transcription 3; BTK, Bruton tyrosine kinase; IRAK4, interleukin 1 receptor associated kinase 4; PROTAC, proteolysis targeting chimeras.

## Author contributions

The first draft of the manuscript was written by XL, WL, and YZ. YS and LX critically revised the manuscript. All authors read and approved the final manuscript.

## Conflict of interests

The authors declare that they have no competing interests.

## Data availability

Data sharing is not applicable to this article as no datasets were generated or analyzed during the current study.
